# Upside-down swimming behaviour of free-ranging narwhals

**DOI:** 10.1186/1472-6785-7-14

**Published:** 2007-11-19

**Authors:** Rune Dietz, Ari D Shapiro, Mehdi Bakhtiari, Jack Orr, Peter L Tyack, Pierre Richard, Ida Grønborg Eskesen, Greg Marshall

**Affiliations:** 1 Department of Arctic Environment, National Environmental Research Institute, University of Aarhus, Frederiksborgvej 399, Postbox 358, DK-4000 Roskilde, Denmark; 2 Biology Department, Woods Hole Oceanographic Institution, MS #50, Woods Hole, MA 02543, USA; 3 National Geographic Remote Imaging, 1145 17th St. NW, Washington, DC 20036, USA; 4 Department of Fisheries and Oceans, Central and Arctic Region, Arctic Research Division, 501 University Crescent, Winnipeg, Manitoba R3T 2N6, Canada

## Abstract

**Background:**

Free-ranging narwhals (*Monodon monoceros*) were instrumented in Admiralty Inlet, Canada with both satellite tags to study migration and stock separation and short-term, high-resolution digital archival tags to explore diving and feeding behaviour. Three narwhals were equipped with an underwater camera pod (Crittercam), another individual was equipped with a digital archival tag (DTAG), and a fifth with both units during August 2003 and 2004.

**Results:**

Crittercam footage indicated that of the combined 286 minutes of recordings, 12% of the time was spent along the bottom. When the bottom was visible in the camera footage, the narwhals were oriented upside-down 80% of the time (range: 61 100%). The DTAG data (14.6 hours of recordings) revealed that during time spent below the surface, the two tagged narwhals were supine an average of 13% (range: 9–18%) of the time. Roughly 70% of this time spent in a supine posture occurred during the descent.

**Conclusion:**

Possible reasons for this upside-down swimming behaviour are discussed. No preference for a clockwise or counter-clockwise direction of roll was observed, discounting the possibility that rolling movements contribute to the asymmetric left-handed helical turns of the tusk.

## Background

The narwhal (*Monodon monoceros*) is an Arctic odontocete that can grow up to 5 m long. Males possess a tusk that erupts from the upper left jaw [1]. Individuals with two tusks and females with one or two tusks have also been reported but are rare [1]. The narwhal lacks a dorsal fin, but it has a low, irregular ridge 4–5 cm high along the posterior half of the back [1]. Narwhals inhabit the inshore bays and island passages of northeastern Canada, Greenland and Svalbard from July through September [2-5]. In the autumn, as Arctic fjords and bays begin to freeze, narwhals vacate these areas and migrate long distances to their wintering areas, which are further south and in deeper water [6-9]. During the winter and spring (November-June), narwhals frequent areas covered with dense (up to 99%) offshore pack ice e.g., [10,11]. Over the last 13 years, satellite tracking studies have provided information about narwhal seasonal and stock distribution as well as migration routes and dive behaviour [6-9,11-14].

**Figure 1:**
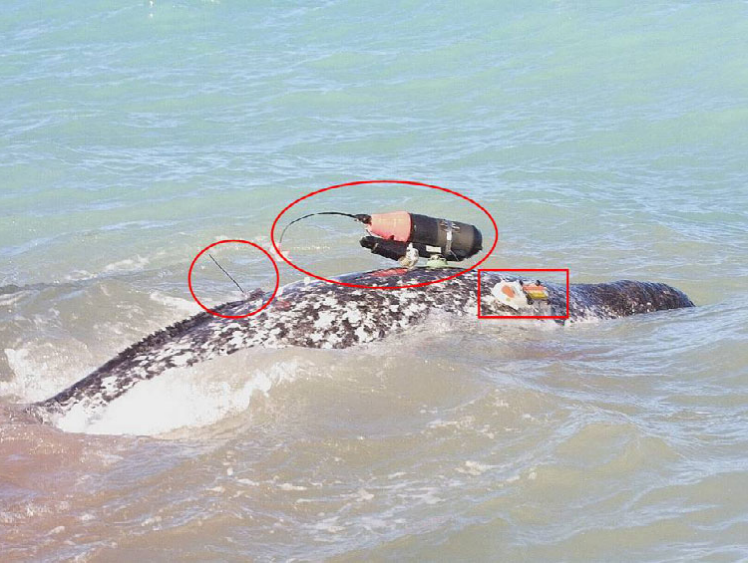
**Narwhal instrumented with satellite, a Crittercam and DTAG tags**. A male narwhal (#37232) tagged with a satellite tag (enclosed by the circle), a Crittercam (enclosed by the ellipse) and a DTAG (enclosed by the rectangle) at Kakiak Point in Admiralty Inlet, Canada on 11 August 2004. Photograph courtesy of Rune Dietz.

Data from satellite-linked time depth recorders (STDRs) and diving archival tags have been used to describe narwhal diving behaviour and derive correction factors for submerged animals when formulating population estimates from aerial surveys [12-15]. Recent advances in non-invasive tagging technologies including the Crittercam and DTAG are offering new insights into the movements of individual, free-ranging whales in their native habitats. The Crittercam (National Geographic Television) was developed to link visual behavioural sequences with other data streams including acoustic, movement and depth information [16]. The DTAG provides high quality acoustic recordings and the depth, orientation and acceleration of the whale to which it has been attached [17]. In this study, access to narwhals handled during the satellite tagging work provided feasible attachment opportunities to deploy the Crittercam and DTAG (Figure 1). We describe and quantify the striking upside-down, or supine, diving behaviour of these animals and discuss why this behaviour may be advantageous to the narwhal. We also examine other finless whales to explore whether they have similar upside-down swimming behaviour.

## Results

### Narwhal orientation in the water column

**Figure 2:**
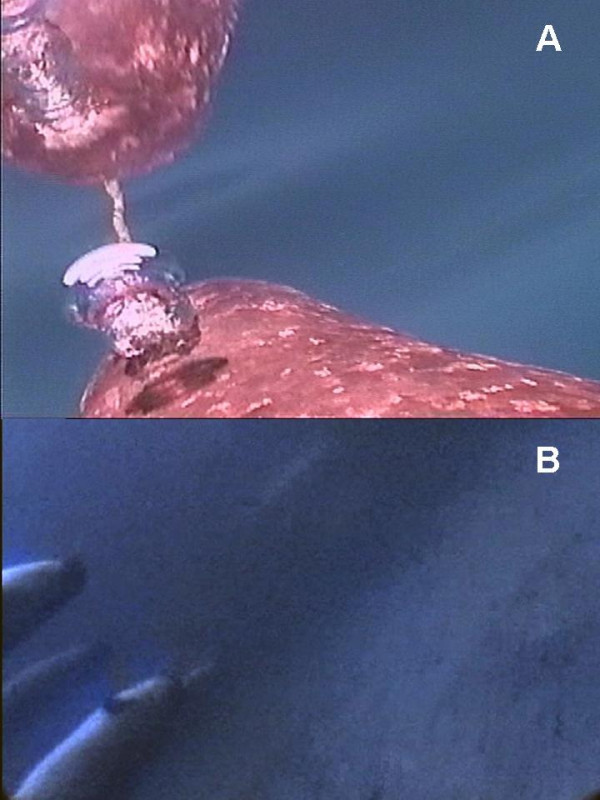
**a Narwhal at surfacing recording from crittercam**. Narwhal starting the blow prior to surfacing at a normal position with the blowhole oriented towards the surface. Note the mirror effect of the water surface. Picture captured from Crittercam footage (see online electronic supplement, additional file 1). **b Narwhal upside-down swimming along the inlet's bottom. **Narwhal swimming upside-down (dark back oriented towards the bottom and the white belly oriented upwards) along the inlet's bottom accompanied by two other narwhals from the same group displaying identical behaviour. Picture captured from Crittercam footage (see online electronic supplement, additonal file 2). Photograph and footage courtesy of National  Geographic Remote Imaging.

The 286 minutes of Crittercam footage revealed that the 4 narwhals spent on average 30% (range: 17–42%) of their time at the surface, 58% (range: 49–69%) in mid-water and 12% (range: 4%–21%) along the bottom. As expected from visual observations in the wild, the whales oriented with their dorsal sides up at the surface (99% of the time) to breathe through their blowholes (Figure 2a, additional file 1). When the tagged narwhals were in the water column where neither surface nor bottom was visible, the orientation could not be determined from the Crittercam data alone. When the bottom was visible on the video recordings, the narwhals were oriented upside-down for 80% of the time (range: 61%–100%; Table 1). This behaviour was also demonstrated when other supine whales appeared in the footage as they swam along the bottom (Figure 2b, additional file 2). A few times the Crittercam actually scraped against the bottom and in one case this caused the camera to release prematurely (this footage was excluded from the analysis here).

**Table 1:** Narwhal orientation results monitored by Crittercam

					Orientation category, % of total time				Orientation category, % of time within each orientation category			
Narwhal #	Sex	Length (cm)	Recording duration (min)	Vertical stratum	Normal	Unknown	Upside-down	Total	Normal	Unknown	Upside-down	Total

3960	F	416	98	Surface	25.9	1.2	0.0	27.1	95.6	4.4	0.0	100.0
				Pelagic	-	69.4	-	69.4	-	100.0	-	100.0
				Bottom	0.2	0.0	3.3	3.5	5.7	0.0	94.3	100.0
				
				Total	26.1	70.6	3.3	100.0	26.1	70.6	3.3	100.0
												
7931	M	349	114	Surface	34.8	0.0	0.0	34.8	100.0	0.0	0.0	100.0
				Pelagic	-	51.9	-	51.9	-	100.0	-	100.0
				Bottom	4.7	0.0	8.5	13.2	35.6	0.0	64.4	100.0
				
				Total	39.5	51.9	8.5	100.0	39.5	51.9	8.5	100.0
												
3964	F	380	40	Surface	17.2	0.0	0.0	17.2	100.0	0.0	0.0	100.0
				Pelagic	0.5	61.4	-	62.0	0.8	99.2	-	100.0
				Bottom	8.1	0.0	12.7	20.8	38.9	0.0	61.1	100.0
				
				Total	25.8	61.4	12.7	100.0	25.8	61.4	12.7	100.0
												
37233	M	415	*34*	Surface	42.3	0.0	0.0	42.3	100.0	0.0	0.0	100.0
				Pelagic	0.0	48.9	0.0	48.9	0.0	100.0	0.0	100.0
				Bottom	0.0	0.0	8.8	8.8	0.0	0.0	100.0	100.0
				
				Total	42.3	48.9	8.8	100.0	42.2	48.8	8.7	100.0
												
Average			286	Surface	30.1	0.3	0.0	30.4	98.9	1.1	0.0	100.0
				Pelagic	0.3	57.9	0.0	58.1	0.4	99.8	0.0	100.0
				Bottom	3.3	0.0	8.3	11.6	20.1	0.0	79.9	100.0
				
				Total	33.4	58.2	8.3	100.0	33.4	58.2	8.3	100.0

Percentage time spent in different orientation categories at different depths based on Crittercam data from four narwhals in Admiralty Inlet in 2003 and 2004.

**Figure 3:**
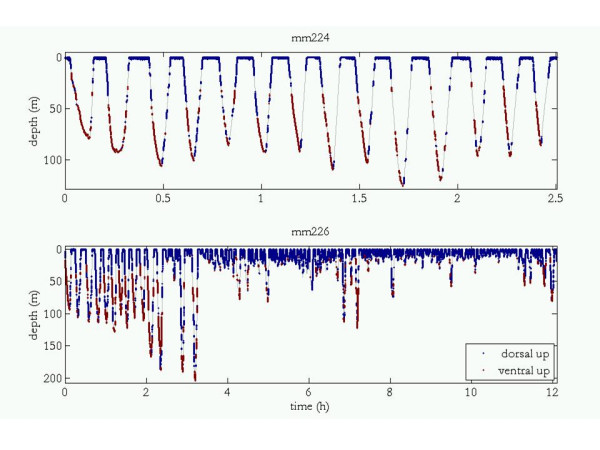
**Narwhal orientation during diving**. Diving record for animals outfitted with DTAG with dorsal and ventral sides up indicated by dark blue and red, respectively (see text for details on their calculation). Portions of the dive with roll values that did not fall into these two categories are grey. Note the different ranges on the time axes.

**Figure 4:**
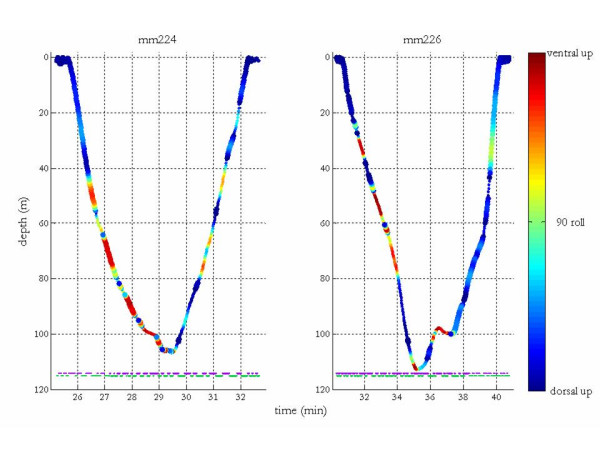
**Another sample figure title**. Roll colour coded (orientation indicated by colour bar) during the third dive of each animal. To dispense with the ambiguity of equal and opposite turns off the central axis, imagine a unit circle coincident with a trans-axial cross-section of the body where 0 and π lie along the right and left sides of the body midline, respectively. The thicker segments in the figure correspond to the roll angle ranging between ±π/2 passing through 0 and the thinner segments to the roll angle ranging between ±π/2 passing through π. The purple and green dots at the bottom of the two panels indicate when the animals were turning clockwise and counter-clockwise, respectively.

The DTAG allowed a more precise quantification of the orientation of the two tagged male narwhals during entire dive sequences (Table 2), but yielded no information about when the animal was swimming along the inlet bottom. A quantitative assessment of the proportion of time spent swimming dorsal versus ventral side up yielded average percentages of 58% vs. 13% (#37232: 53% vs. 18%; #37233: 65% vs. 9%) from the DTAG sample. The remaining 29% of the time the animals had intermediate orientations while moving between the normal or upside-down orientation. The tagged whale was oriented upside-down 70% of the time during the descent period of the dives (#37232: 87%; #37233: 56%) and an average of 77% of the time during the initial 60% of the time of the dive (defined as a vertical excursion exceeding 20 m; #37232: 89%, #37233: 65%). Figure 3 illustrates when the two animals with a DTAG were oriented dorsal or ventral side up for the entire diving sequence. Figure 4 focuses on a single dive of each animal to illustrate their complex roll dynamics. Individual #37232 rotated to a supine orientation and remained upside-down for the majority of the dive between roughly 45 and 105 m before ascending and rolling slightly to spend the last portion of its ascent dorsal side up. Individual #37233 corkscrewed continuously on the descent, rolling repeatedly before reaching a maximum depth of ~113 m. Between recorded times of 36.5 and 37.5 min, his supine orientation coincided with a relatively level depth, suggesting he may have been moving along a flat seabed at that time. His return to the surface was unremarkable in terms of roll as he remained dorsal side up during nearly the entire ascent.

**Table 2:** Narwhal orientation and turning direction monitored by DTAG

		Entire water column		Upper 3 m		% time spent turning	
Animal	Recording duration (h)	Dorsal up	Ventral up	Dorsal up	Ventral up	Clockwise	Counter-clockwise

#37232	2.5	52.9%	18.0%	96.8%	0%	49.7%	50.3%
#37233	12.1	64.5%	9.0%	98.9%	0%	49.2%	50.8%
Average		57.5%	13.3%	97.5%	0%	48.4%	51.6%

Summary of DTAG data from two narwhals tagged in Admiralty Inlet in August 2004 including information on recording longevity, turning direction and orientation through the entire recording period and at the surface (upper 3 m).

In the top 3 m of the water column, these two male narwhals were oriented dorsal side up 98% of the time on average according to the DTAG record (#37232: 97%; #37233: 99%) and were never oriented ventral side up. For narwhal #37233, it was possible to compare the Crittercam recording to the first 25 minutes of data gathered by the DTAG to investigate how often the bottom appeared in the frame when the animal was swimming upside-down. During this single period of instrumentation overlap across all animals, the DTAG recorded that the narwhal was upside-down 28% of the time compared to the Crittercam footage reporting a value of 9%. These numbers demonstrated that the animals were oriented upside-down during periods of time when the bottom was not visible in the Crittercam footage. This suggests that the narwhals may have assumed a supine posture mainly as they descended but also as they swam and ascended independently of whether they were very near to the bottom.

### Narwhal rolling direction

The Crittercam footage contained 20 instances where the direction of change in roll could be distinguished. Exactly half of these changes were clockwise and half were counter-clockwise. Clockwise and counter-clockwise changes in the direction of roll computed from the DTAG data for the representative dives of the two tagged animals are indicated at the bottom of Figure 4. The DTAG records similarly revealed no evident bias in the turning direction for either animal (chi-square test was not significant for any of the entries in Table 2; Figure 4).

## Discussion

As the results are based on information from 6 narwhals outfitted with Crittercam and DTAGs, the findings and discussions are based on a relatively small sample size and should be interpreted cautiously.

### Narwhal orientation in the water column

The Crittercam and DTAG recordings revealed that the tagged narwhals spent a substantial period of time oriented in a supine posture as they swam. This was documented both by the orientation data from the tagged whales and by video footage of other whales that were swimming ventral side up along the sandy and flat bottom. The extent to which all supine time periods recorded by the DTAG corresponded to intervals spent near or along the bottom could not be determined, but the short segment of overlapping video and DTAG data suggested that this behaviour occurred independently of proximity to the bottom.

It is not evident why narwhals display this behavioural tendency of swimming upside-down as they dive. The orientation may relate to the transmission beam pattern of their echolocation clicks. The beam pattern of narwhal clicks has not been measured but the beam of the most closely related species, the beluga whale (*Delphinapterus leucas*), was oriented about 5 degrees above the plane defined by the animal's teeth [18]. The orientation of the melon towards the bottom may direct the sonar beam downwards where the prey is likely to be most abundant. However, no convincing acoustic evidence supports this hypothesis since echolocation indicative of foraging occurred only rarely in the corresponding acoustic recordings during this upside-down orientation and feeding was not observed on the Crittercam footage [see [19]].

The absence of a pronounced dorsal fin and its replacement with a shallower dorsal ridge allows the animal to manoeuvre more easily ventral side up closer to the inlet bottom. It is believed that this lack of a dorsal fin has evolved as an adaptation to navigate beneath ice-covered waters [1]. This explanation has been proposed for the absence of a dorsal fin among two other cetacean species that are also associated with dense pack ice: the beluga and the bowhead (*Balaena mysticetus*) whales [20,21]. However, other species including the sperm whale (*Physeter macrocephalus*) described as having a "low, thick, and rounded or obtuse" dorsal fin and the finless porpoise (*Neophocaena phocaenoides*) and northern and southern right whale dolphins (*Lissodelphis borealis and L. peronii*, respectively) that lack pronounced dorsal fins do not always live in ice-covered environments [22-24]. According to Fish [25], the cetacean dorsal fin is used to provide balance, stability and maneuverability. In addition, the lack of a dorsal fin favours a flexible body. Cetaceans with such flexible body designs (e.g., *Delphinapterus*, *Inia*) hence sacrifice speed for manoeuvrability [25]. Given this discrepancy, it is possible that the dorsal ridge of the narwhal and these other cetaceans may facilitate certain aspects of their underwater movement behaviour including roll.

The distinctive tusk that is characteristic of the majority of subadult and adult males and the occasional female may also relate to the upside-down behaviour observed here. If an animal close to the bottom were oriented dorsal side up and attempted to bend its head downwards to orient its melon towards the bottom, the tusk would be in danger of hitting the ground. The large proportion of broken tusks (34%) from a large West Greenland sample suggests that the tusk is brittle [26,27], and that the narwhals should be cautious of breaking their tusks. The upside-down orientation would allow the tusk to be positioned near the bottom to scare and subsequently guide demersal prey towards its mouth like a shovel. The use of the tusk in association with feeding has been suggested previously [27,28], proposing that the tusk was used to uncover and root out prey along the bottom. The tusk is often worn down at the tip, suggesting that it occasionally has a less severe contact with softer bottom substrate (e.g. mud, sand or gravel). Scarring on the head and broken tusks have been linked to violent fighting between sexually mature males [29,30]. However, some scarring of the head could also be a consequence of swimming upside-down close to the seabed. Female narwhals and immature males also have some head scars, albeit not quite as many as mature males [30]. Wounds from such injuries can heal, which is not the case for a broken tusk. Finally, the tusk, although oriented along the longitudinal axis of the body, is angled slightly downward [31]. The downward angle is believed to have evolved to reduce the likelihood of the tusk being damaged when swimming under ice. However, near the bottom, it therefore may be better for the narwhal to swim upside-down.

If the narwhal swam flat against the bottom with its dorsal side up, it would have the advantage of having its mouth closer to benthic prey than if it were upside-down. However, the lower jaw of a narwhal is fragile because it is hollow and thin-boned probably because it is used for sound reception, as suggested by Norris [32] and Norris & Harvey [33] for other odontocetes. An open jaw hitting a hard bottom at a speed of 12 m/s could cause substantial damage. Hence, protection of the jaw could be another reason for the upside-down swimming.

On the Crittercam footage, we observed supine behaviour among both male and females, suggesting that the explanations relating to the use of the sonar and a protection of the fragile lower jaw may be most relevant for both sexes. The role of the tusk in feeding cannot be obligatory since female groups are often segregated from the males during a large part of the year but are still able to obtain food [34].

This supine swimming behaviour has important implications for the longevity and durability of tags deployed on both the tusk and dorsal surface of a narwhal. Premature failure of such equipment may be explained by transmitter collisions with the bottom, a danger also associated with swimming in ice-covered waters.

### Upside-down swimming in other whale species

Some attention has been paid in the literature to upside-down swimming in other whale species. Akamatsu et al. [22] reported observations of finless porpoises swimming upside-down but did not quantify that behaviour or determine whether it was related to depth and proximity with the seabed. The Amazon river dolphin (*Inia geoffrensis*) has been reported to sleep underwater in an upside-down orientation [35]. Fristrup & Harbison [36] presented two hypotheses about the function of the rolling behaviour in the context of prey detection and capture by sperm whales. They postulated that sperm whales locate their prey visually, either silhouetted against down-welling daylight or by scanning for bioluminescence produced by the movements of their prey. However, results from DTAGs on sperm whales in the Mediterranean Sea and Gulf of Mexico do not support either hypothesis [37]. No consistent upside-down behaviour in the roll data is evident when creaking and sperm whales generally feed at depths without light from the surface. The acceleration bursts measured in the DTAG data also contradict the expectations of the second hypothesis [37]. Miller et al. [37] reported that sperm whales actively altered their body orientation throughout the bottom phase of their dives with significantly increased manoeuvring while producing creaking sounds. The creaking sounds are thought to be bursts of echolocation pulses used for the final stage in a foraging sequence. Grey whales and some river dolphins also feed by turning sideways near the bottom. Woodward & Winn [38] used a DTAG to demonstrate that a feeding grey whale spent more than half of its bottom time rolled at an angle >45°. The Indus river dolphin, (*Platanista minor*) is reported to swim on its side near the bottom of the muddy river, echolocating more or less continuously [39].

### The turning direction relative to the turning of the tusk

No clear preference for turning direction was found for any of the tagged animals. There has been speculation surrounding the left-sided helical geometry of the narwhal tusk, how it has evolved and whether its conformation is influenced at all by the behaviour of the animal [40-42]. If narwhals turned consistently in a clockwise direction, this biased movement might support such asymmetric growth. However, neither the Crittercam nor the DTAG revealed such a preference in rolling direction (i.e., clockwise or counter-clockwise), casting serious doubt on such an explanation. In contrast, the study on grey whales by Woodward & Winn [38] using DTAGs documented that 97% of these rolls were clockwise. This matches a report from Kasuya & Rice [43] indicating that the right side of the grey whale rostrum had fewer barnacles and more scrapes than the left side. They also reported shorter baleen on the right side than on the left in 28 of the 31 animals measured, suggesting that most grey whales roll to the right when foraging.

## Conclusion

Our results describe a characteristic underwater supine swimming behaviour of narwhals and similar behaviour in other species as well. We have ventured some possible explanations for its function and conclude that the most likely explanations relate to an improved use of the sonar and possibly to a protection of the fragile lower jaw as well. Further research is required on narwhals, the closely related beluga and other species with and without dorsal fins to determine whether upside-down rolling influences foraging and other behaviours.

## Methods

The long-term Canadian high Arctic narwhal tracking project conducted by the National Environmental Research Institute in Denmark, the Greenland Institute of Natural Resources and Canada's Department of Fisheries and Oceans provided an opportunity to attach Crittercams and/or DTAGs to narwhals during two summer field seasons in Admiralty Inlet in 2003 and 2004.

### Field site

The whales were tagged at Kakiak Point (72.682° N; 86.687° W), Admiralty Inlet, Baffin Island, Canada. This fjord serves as a habitat for thousands of narwhals during August and early September. Admiralty Inlet is a fjord extending up to 40 km in width and 300 km in length with a maximum depth of roughly 700 m. The fjord is bordered by high (200–500 m), steep-sided mountains of the Brodeur Peninsula Plateau to the west and the Borden Peninsula to the east [44]. The shores of Admiralty Inlet offer promontories of low bluffs or raised beaches including our field site at Kakiak Point. Field work was conducted from 16–21 August 2003 and 11–22 August 2004.

### Netting and handling of the whales

The narwhals were captured in nets set close to the surface and perpendicular to the shoreline [6-9]. Due to the large number of whales in the area, only one 50 m long section of net mounted approximately 50 m from the shore was set on the north side of the peninsula to avoid capturing too many individuals simultaneously [45]. The nets were kept under continuous surveillance so the team could react rapidly to any entangled narwhals. Immediately after a whale became entangled, two inflatable boats were dispatched to secure the captured animal. Nets and the narwhal were first raised to the surface to ensure that the captured animal could breathe freely. Once taken to shore and secured by a tail rope, the remaining net was cut off the whale. A hoop net was used to support the head and hold the blowhole above water. A satellite transmitter (STDR) was then surgically attached to the animal's dorsal ridge. Immediately before its release, the Crittercam and/or DTAG were/was attached by suction cups near the dorsal ridge. After instrumentation, the narwhals were released into deeper water.

### The tagged whales

Thirteen and eight narwhals were satellite tagged in 2003 and 2004, respectively [45]. Of these 21 animals, eight were tagged with a Crittercam over the two years and three were fitted with a DTAG in 2004. Only four (two males and two females) Crittercam deployments provided usable video recordings; the others did not provide data due to premature release (once) or lack of retrieval (three). Two of the 3 DTAG deployments were recovered for data extraction (Table 3). The Crittercams yielded a total of 4.8 h of footage and the DTAGs provided 14.6 h of recordings. Only one narwhal allowed simultaneous recordings from a Crittercam and a DTAG, an overlap which lasted about 25 minutes (Figure 1).

**Table 3:** Basic information on the tagged animals

**Year/Transmitter#**	**DTAG ID**	**Date**	**Sex**	**Length**	**Fluke width**	**Tusk length**	**CC footage**	**CC TDR-data**	**DTAG**
***2003***									
3960		16 Aug.	Female	416	?	-	+	-	-
37235		18 Aug.	Male	448	104	193	-	+	-
7931		19 Aug.	Male	349	90	68	+	+	-
3964		21 Aug.	Female	380	88	-	+	+	-
***2004***									
37232	mm224	11 Aug.	Male	392	100	202	-	-	+
37233	mm226	13 Aug.	Male	415	105	165	+	+	+

Narwhals equipped with Crittercams (CC) and DTAGs in Admiralty Inlet in August 2003 and 2004 with tagging date and basic biological parameters indicated. A '+' indicates successful acquisition of a dataset whereas a '-' indicates an unsuccessful or un-attempted acquisition.

### Crittercam and DTAG specifications

A Crittercam is composed of a water- and pressure-proof housing and contains either a mini digital video camera (length: 25.4 cm, diameter: 8.9 cm and weight: 1.8 kg in air) or a Hi-8 camera (length: 30.5 cm, diameter: 10.2 cm and weight: 2 kg in air). The Crittercam was mounted via suction cup attachment with a programmable release mechanism (Figure 1) and was capable of recording up to 3 hours of video, audio, pressure and temperature data before release. The DTAG (10.2 cm × 3.8 cm × 2.3 cm; weight: 133 g in air) sampled movement at a frequency of 50 Hz and an audio frequency of 96 kHz [see [16]]. The unit was attached with 4 suction cups and a release mechanism that was coupled to the release of the Crittercam (Figure 1). Once the tags released and floated to the surface, their onboard VHF beacons were used for recovery of the units using an Inuit-operated boat and a handheld Yagi antenna [e.g., [13]].

### Data analysis

Video footage from the Crittercam was visually inspected once each second to log both the orientation of the narwhal if the water surface or seafloor was visible (3 categories: dorsal side up; ventral side up; unknown) and its position in the water column (3 categories: at or near the surface when the surface was visible in the frame; mid-water when neither the surface or the bottom was visible; along the bottom whenever visible). When it could be distinguished, the direction of the roll (clockwise or counter-clockwise) was also noted.

All DTAG sensors were calibrated using laboratory-derived constants adjusted to the specific conditions of each tag recording to minimize temperature- and pressure-related sensor offsets, the resulting data were decimated to 5 Hz and a pitch, roll and heading file describing each animal's orientation was generated [see [16]]. The movement records from the DTAG allowed a similar appraisal of dorsal and ventral up orientation defined as within 45° of these absolute orientations. In other words, we imagined a unit circle coincident with a trans-axial cross-section of the body where 0 and π lay along the right and left sides of the body midline, respectively. Dorsal and ventral sides up corresponded to the ranges [3π/8, 5π/8] and [-3π/8, -5π/8], respectively. The clockwise or counter-clockwise turning direction during roll could also be determined from the DTAG. After the roll data were decimated to a sampling rate of 1 Hz and filtered using a 5-point running average, their derivative was taken to provide a continuous description of the direction of roll (i.e., clockwise vs. counter-clockwise) for the duration of the tag recordings. A chi-square test with 1 degree of freedom was used to test for significant deviation in the roll direction from an expected even split between clockwise and counter-clockwise turning. The descent portion of each dive was defined as the interval between the fluke out immediately following the span of time spent at the surface and the first period when the animal was pitched level or at a positive deflection from the horizontal for at least 5 s.

## Authors' contributions

RD organized the field work, conceived of the study, helped analyze the data and drafted the manuscript. ADS participated in the field work, analyzed the data and helped draft the manuscript. MB was engineer for the Crittercam, participated in the field work and made data available for the study. JO principally organized and participated in the field work. PLT co-developed the DTAG, facilitated the collaboration of the co-authors and helped draft the manuscript. PR helped organize the field work. IGE extracted data from the Crittercam recordings. GM initiated and led the Crittercam project and made data available for the study. All authors read and approved the final manuscript.

## Supplementary Material

Additional file 1 Crittercam AVI footage of a surfacing narwhal for internet version of the article (Narwhal surfacing.avi). Click here for file

Additional file 2 Crittercam AVI footage of the upside-down swimming behaviour along the bottom of a group of narwhals for internet version of the article (Narwhal upside-down swimming bottom.avi). Click here for file
